# Olfactory Attraction of the Larval Parasitoid, *Hyposoter horticola*, to Plants Infested with Eggs of the Host Butterfly, *Melitaea cinxia*

**DOI:** 10.1673/031.010.5301

**Published:** 2010-06-04

**Authors:** Marcela K. Castelo, Saskya van Nouhuys, Juan C. Corley

**Affiliations:** ^1^ CONICET - Grupo de Investigación en Ecofisiología de Parasitoides - Departamento de Ecología, Genética y Evolución - Facultad de Ciencias Exactas y Naturales - Universidad de Buenos Aires, Ciudad Universitaria, Pabellón II, (CI428EHA) Ciudad de Buenos Aires, Argentina.; ^2^ Department of Biological and Environmental Sciences, Division of Population Biology, PO Box 65, FIN, University of Helsinki, Finland and Department of Ecology and Evolutionary Biology, Cornell University, Ithaca, NY, USA.; ^3^ CONICET - Laboratorio de Ecología de Insectos - INTA Bariloche, (R8400HRG) Bariloche, Río Negro, Argentina.

**Keywords:** chemical cues, foraging behavior, insect-plant interactions, multitrophic level interaction, *Plantago lanceolata*, *Veronica spicata*

## Abstract

Parasitoids locate inconspicuous hosts in a heterogeneous habitat using plant volatiles, some of which are induced by the hosts. *Hyposoter horticola* Gravenhost (Hymenoptera: Ichneumonidae) is a parasitoid of the Glanville fritillary butterfly *Melitaea cinxia* L. (Lepidoptera: Nymphalidae). *Melitaea cinxia* lays eggs in clusters on leaves of *Plantago lanceolata* L. (Lamiales: Plantaginaceae) and *Veronica spicata* L. (Lamiales: Plantaginaceae). The parasitoid oviposits into host larvae that have not yet hatched from the egg. Thus, though *H. horticola* is a parasitoid of *Melitaea cinxia* larvae, it must find host eggs on plants that have not been fed on by the larvae. Using a Y-tube olfactometer, the response of *H. horticola* to odors of *Melitaea cinxia* and extracts of the attacked plant species were tested. Three week-old eggs (near hatching) were attractive to young *H. horticola*, but one week-old eggs were attractive only to old or experienced *H. horticola*. *Melitaea cinxia* larvae were not attractive. A water extract of *P. lanceolata* was attractive, but ethanol or hexane extracts were not. None of the extracts of *V. spicata* were attractive. Leaves of *V. spicata* were attractive only if harboring eggs, but *P. lanceolata* leaves with eggs were not. Free flying *H. horticola* in a large outdoor enclosure were presented with host and plant cues. As in the olfactometer, *V. spicata* was attractive only when eggs were on it, and *P. lanceolata* was somewhat attractive with or without eggs. This study shows for the first time that a parasitoid of larvae uses egg volatiles or oviposition-induced plant volatiles, to find host larvae, and that *Melitaea cinxia* eggs or traces of oviposition induce the production of these volatiles by the plant. Based on the results, and given the natural distribution of the plants and *M. cinxia* eggs, parasitism of *Melitaea cinxia* eggs on *P. lanceolata* would be expected to be low. Instead, under natural conditions, a fraction of the eggs in virtually all egg clusters are parasitized on both plant species. The mismatch between the experimental results and the natural pattern of host-parasitoid interactions is discussed in terms of the expected coupling foraging cues with foraging success.

## Introduction

Parasitoids find hosts by responding to cues from their surroundings. A good cue reliably signals the presence and quality of a host and is detectable over an appropriate distance (Vet and Dicke 1992; Hilker and McNeil 2007). These cues are, to a large extent, volatile odors derived from the host or from the host plant as a result of injury or the presence of saliva triggering production of attractive volatiles (Turlings et al. 1995; Felton and Eichenseer 1999; Kessler and Baldwin 2001). Herbivore eggs cause little or no damage to plants, so egg parasitoids must use indirect cues while foraging (Hilker and Meiners 2006; Fatouros et al. 2008b). Where eggs are closely associated with herbivory, egg parasitoids can use herbivore-associated odor cues. For example, bean plants with oviposition and feeding by the pentatomid bug, *Nezara viridula* produced volatiles that attract the egg parasitoid *Trissolcus basalis* (Colazza et al. 2004). Plant odors alone can also be used by egg parasitoids (Romeis et al. 2005), as is the case for *Platygaster demades*, which is attracted to the odors of apple and pear foliage even without signs of host activity (Sandanayaka and Charles 2006). However, because most plant individuals do not have eggs on them, plant odor alone is an unreliable cue. Some egg parasitoids respond to odors of adult hosts (Noldus et al. 1991; Conti et al. 2003; Fatouros et al. 2007) such as moth scales, marking pheromones, and sex pheromones that are deposited on plants or eggs during oviposition (i.e., DeLury et al. 1999). Finally, host oviposition can induce the plant's emission of volatiles that are attractive to parasitoids. Plants have been shown to respond in various ways to damage caused by oviposition or to chemical recognition of the surface of the eggs or adhesive. A literature review by Hilker and Meiners (2006) identified three studies of plants that produce volatiles that are attractive to parasitoids in response to oviposition including the elm leaf beetle on elm (Meiners and Hilker 2000), the pine sawfly on pine (Hilker et al. 2002) and Hemiptera on bean (Colazza et al. 2004). No Lepidoptera have been found to induce volatile odors by oviposition, though the cabbage white butterfly does cause a local change in surface chemistry that arrests parasitoid foraging behavior (Fatouros et al. 2005).

Whatever the cues, over time, parasitoid response changes. This can be due to parasitoid age or physiological state (i.e., Amalin et al. 2005; Crespo and Castelo 2008). For instance, the patch residence time for the parasitoid *Lysiphlebus cardui* increases with parasitoid age, and younger parasitoids lay more eggs in second and third instars of the host, while older parasitoids show no preference (Weisser 1994). Independent of age, parasitoid response to cues also changes with experience, especially due to learning in association with positive foraging experience (i.e. Bellows 1985; van Baaren and Boivin 1998; for review see Papaj and Lewis 1993).

*Hyposoter horticola* Gravenhost (Hymenoptera: Ichneumonidae) is a parasitoid of the Glanville fritillary butterfly *Melitaea cinxia* L. (Lepidoptera: Nymphalidae). In the Åland islands of southwest Finland, *Melitaea cinxia* lays egg in clusters on the undersides of leaves of two plant species, *Plantago lanceolata* L. (Lamiales: Plantaginaceae) and *Veronica spicata* L. (Lamiales: Plantaginaceae) (Kuussaari et al. 2004). *M. cinxia* spends up to an hour ovipositing a cluster of eggs. During that time, it touches the leaf with its tarsi and rubs the underside of the leaf with the ovipositor. *Melitaea cinxia* also attaches the eggs to the leaf with an adhesive substance (Singer 2004). Although *Hyposoter horticola* is a parasitoid of larvae, it must find the hosts as eggs because it oviposits into host larvae that have not yet broken out of the eggshell. The host can only be used as a larva inside the egg, so as eggs get older, they get closer to the interval when they can be parasitized. *H. horticola* finds egg clusters during the two to three weeks before hatching, and monitors them until the eggs are briefly suitable for oviposition (van Nouhuys and Ehrnsten 2004). The vast majority of the *M. cinxia* egg clusters are on undamaged plants. Based on landscape scale studies of this host-parasitoid interaction, virtually all of the host egg clusters, under natural conditions, are found by the parasitoids (and a fraction of the hosts in each cluster are parasitized), regardless of which plant species they are on and regardless of where they are in the landscape (van Nouhuys and Hanski 2002; van Nouhuys and Kaartinen 2008).

This report presents a set of experiments addressing the host-finding cues used by *H. horticola*. Young, medium, and old eggs, as well as host larvae and host plants, were tested to determine whether they emit volatiles that are attractive to *H. horticola* under laboratory conditions using a Y-tube olfactometer. Outside, in a field cage experiment at the scale of a habitat patch, the ability of parasitoids to find the eggs, using the same cues found to be important in the laboratory tests, was tested.

The rationale for the field experiment stemmed from the observation that, while host eggs on *V. spicata* and *P. lanceolata* are both used quite successfully in the field, they elicit different responses from *H. horticola* in the olfactometer. Cues identified as attractive in an olfactometer are expected to correspond to cues used naturally in the field (recent examples include Lou et al. 2006; Dormont et al. 2007; Zahng et al. 2007). However, this is not always the case (i.e. Ngumbi et al. 2005), perhaps because in the field an attractive compound may be at low concentration or simply not perceived in a more complex chemical environment (Hilker and McNiel 2007). Furthermore, additional cues may be present in the field, both visual and olfactory, that lead *H. horticola* to a different destination.

## Materials and Methods

### Hosts, plants, and parasitoids

For both the laboratory experiment (2004) and the field experiment (2006), parasitoids were obtained by placing laboratory-reared host egg clusters in natural populations of the host butterfly *M. cinxia* in Åland, Finland, the summer before each experiment. Eggs on plants were obtained as described below. When the egg cluster was 7 to 14-days-old, the infested plant was introduced in the field. After parasitism in the field, the infested plant, now with larvae instead of eggs, was retrieved, and the larvae were reared through a winter diapause until pupation the following spring. After emergence, adult *H. horticola* were fed a 1:3 honey:water solution and kept individually in plastic vials in a cool environment (9–11° C) until used.

The host egg clusters used to collect *H. horticola* from the field (above), and used for both the olfactometer and field experiments, were obtained using laboratory-reared mated female *Melitaea cinxia* originating from the Åland islands. *M. cinxia* were put individually in outdoor oviposition cages with potted *V. spicata* and *P. lanceolata* plants. The plants were grown outdoors in pots from field-collected seedlings. After one day, the plant with an egg cluster on it was removed and replaced with an new plant. For the olfactometer experiment testing egg odor alone, the leaf with the eggs on it was cut from the plant after oviposition. When the leaf and eggs had dried, the eggs were removed with a tiny brush and placed in a filter paper cup. The egg clusters then were kept individually in Petri dishes in a growth chamber at a temperature of 11° C at night and 22° C during the day. For tests of plants with egg on them, the eggs were left on the plant, and the potted plants with eggs on them were kept under the same conditions as the eggs alone.

### Olfactometer experiments: Tested odor sources in olfactometer experiments

To evaluate the response of *H. horticola* to the odor of host eggs, host larvae, and host food plants, the behavior of adult females was observed using a Y-tube olfactometer. Similar devices have been used to measure behavioral responses of many parasitoid species and mites to odor sources (Janssen et al. 1995; Castelo et al. 2003; Colazza et al. 2004; Martínez et al. 2006). The olfactometer was a 20 cm Y-shaped glass tube connected to an air pump at one end and a plastic box that contains an odor source at the end of each Y-arm. Air was drawn through a carbon filter, and then from the arms of the tube toward its base. The speed of incoming air in each arm was maintained at a constant 0.7 cm/s throughout the experiments. To eliminate possible effects of visual cues on parasitoid behavior, the walls of the odor source-containing boxes were covered, so *Hyposoter horticola* could smell, but not see, the stimuli source. The entire olfactometer was in a white plastic box (50 × 40 × 25 cm) that was open at the top. This allowed *H. horticola* to move within a visually symmetrical environment and reduced disturbances caused by the observer's presence. All trials were conducted between 10:00 and 17:00 h.

Before the experiments, *H. horticola* were removed from the cold, fed honey and water, and kept at ambient temperature for two hours, when they became fully active. *H. horticola* were categorized as young (from 15 to 20 days) or old (from 26 to 33 days). In natural populations *H. horticola* live at least 5 weeks (van Nouhuys and Ehrnsten 2004). Sixty-eight unmated female parasitoids without oviposition experience were used. Unmated parasitoids were used because of the difficulty in making them mate in the laboratory. Although mating status could influence their behavior, *H. horticola* were generally responsive to foraging cues, and there was no reason to believe that their virginity biased their behavior. Because of the limited number of *H. horticola* available, they were used multiple times. For each trial, the parasitoid was chosen randomly from the 68 available parasitoids. Because many experimental trials were performed, each individual was used in an average of eight different trials, randomly spaced among experimental days. This procedure allowed us to perform many trials. However, each parasitoid had a different history of experience, and any effect of age could not be separated from the effect of general odor experience. *Hyposoter horticola* were housed individually, and each had an individual identification number (“wasp ID” in analysis).

### Host eggs and larvae

In this experiment, young and old *H. horticola* were offered intact *M. cinxia* egg clusters of different ages as follows: (a) 1 week-old eggs — young *H. horticola* (*n* = 30), (b) 1 week-old eggs — old *H. horticola* (*n* = 30), (c) 2 week-old eggs — young *H. horticola* (*n* = 53), (d) 2 week-old eggs — old *H. horticola* (*n* = 30), (e) 3-week-old eggs — young *H. horticola* (*n* = 26), and (f) 3 week-old eggs — old *H. horticola* (*n* = 15). The egg clusters contained 100 to 150 eggs. There was no way to count the number of eggs, but by visual estimation it was determined that egg cluster size was not associated with egg cluster age. The order of the treatments was randomized, so in each experiment, *H. horticola* had different previous experience in the olfactometer. In each test, the eggs were placed on a piece of clean filter paper in one arm of the olfactometer, and the other arm contained only clean filter paper.

### Extracts from uninfested plants

In this set of experiments, the parasitoid response to components of plant odor was tested. Leaves of *P. lanceolata* and *V. spicata* were gathered fresh from local, natural populations in the Åland islands. Distilled water, ethanol and hexane extracts of each plant were used as odor sources, and clean solvent was used as the control. Plant solutions were made by grinding 10 g of leaf in 50 ml solvent (200 mg/ml). Extracts and solvent were presented to *H. horticola* as saturated filter paper patches (2 × 2 cm). For each assay, the patches were replaced (one patch-pair per parasitoid), and the side of the Y-tube containing the odor sources was alternated. The treatments were as follows: (a) *P. lanceolata* hexane extract (*n* = 45), (b) *V. spicata* hexane extract (*n* = 45), (c) *P. lanceolata* ethanol extract (*n* = 45), (d) *V. spicata* ethanol extract (*n* = 45), (e) *P. lanceolata* water extract (*n* = 52), and (f) *V. spicata* water extract (*n* = 52). The order of the treatments was randomized, and both young and old *H. horticola* were used for each treatment.

### Host egg-infested plants

To test whether leaves with *M. cinxia* eggs on them were attractive to *H. horticola*, leaves of *P. lanceolata* and *V. spicata* harboring *M. cinxia* egg clusters were placed in the odor source chamber, and clean leaves were used as controls. The leaves with eggs on them were cut off the plant just prior to use, and the control leaves were taken from an eggless plant. The egg clusters each contained 100 to 150 eggs, which did not appear to differ between plant species. Because the number of egg clusters on plants was limited, one egg cluster was used for five to 10 wasps. Again, the position of the odor sources was alternated between assays. *H. horticola* were presented with the following treatments: (a) *P. lanceolata* with eggs (5, 8, and 16) (*N* = 31), and (b) *V. spicata* with eggs (9 and 16) (*n* = 31). There were no young (5 day-old) eggs on *V. spicata* available at the time of the experiment.

### Field cage experiments with free-flying *H. horticola*

*H. horticola* were observed foraging for eggs in a large semi-natural outdoor enclosure, in order to elucidate which odor cues might be used successfully in the field. There were seven treatments: *P. lanceolata* with no eggs (P), *V. spicata* with no eggs (V), *P. lanceolata* with *M. cinxia* eggs (PE), *V. spicata* with eggs (VE), each plant species with eggs 5 cm from the plant (P+E and V+E), and a pot with soil and host eggs but no plant (E). For the eggs alone treatment (E) and eggs near plant treatments (P+E and V+E), a cluster of eggs was gently transferred from a plant into a 1 × 1 cm open filter paper cup, and placed on bare soil in a pot. The egg clusters contained 100 to 150 eggs. Relatively old eggs (19 to 22 days) were used because at this age *H. horticola* were extremely interested in the eggs once they locate them. Upon encountering eggs, *H. horticola* attended to them for approximately 3 to 30 minutes, even if the eggs are not ready for parasitism. This behavior allowed reliable observation of *H. horticola* visiting the egg clusters (van Nouhuys and Ehrnsten 2004).

The experiment took place in a 26 × 32 m mesh-enclosed habitat patch. There were abundant naturally occurring nectar flowers for *H. horticola* to feed on, but there were no naturally occurring hosts or host food plants. The enclosure was previously used for behavioral experiments using *M. cinxia* (Hanski et al. 2006) and *H. horticola* (van Nouhuys and Kaartinen 2008). In each of two trials, there were two replicates of each treatment except the eggs alone (E), which was replicated four times, for a total of 16 observation points. The 16 observation points were set in a randomized grid, 5 × 6 m apart. Several days before the experiment, 23- to 30-day-old unmated adult female *H. horticola* were individually marked on the back of the thorax using craft paint. In order to do this, they were briefly anesthetized using CO2 gas. Twenty-two individually-marked females were released in the cage at 09:00 (while the cage was in the morning shade, and thus they were not active). Then each of the 16 observation points was observed by walking in a transect, every half hour during *H. horticola* foraging hours (09:30–18:00) for two days. The transect walker recorded the number and identity of the parasitoids found at each observation point. For the second trial, a new set of plants and eggs were placed in a re-randomized grid. A second set of 22 individually-marked females was released at 09:00, and the transect was walked every half hour for one day. No observations of *H. horticola* at the observation points were made on the second day of the second trial because most of the parasitoids disappeared, probably due to predation by an extremely large population of spiders inhabiting the grass and mesh walls of the cage.

### Statistical analysis

For the eggs alone and plant extract olfactometer experiments (experiments 1 and 2) the proportion of *H. horticola* that went toward a given odor source was analyzed using Chi-square tests. In order to analyze the response of *H. horticola* to plants with eggs on them (experiment 3), taking into account potential variation due to *H. horticola* age, *H. horticola* experience, egg age, and the day of the trial, a logistic regression analysis was performed with egg age (1, 2, or 3 weeks-old), plant species (*P. lanceolata* or *V. spicata*), date of trial, and *H. horticola* age as factors. Wasp ID was included in the model as an offset covariate because each parasitoid was used more than once (*H. horticola* was chosen randomly from the pool of 68). Date of trial was included because *H. horticola* behavior is affected by ambient temperature and light, which differed daily. The binary dependent variable took a value of 1 when *H. horticola* walked toward the eggs and 0 when *H. horticola* walked toward the control.

For the free foraging experiment, the results from the two trials were combined because there was a small amount of data. A Poisson regression was performed on the counts of parasitoids visiting each treatment-type (PE, VE, P+E, V+E or E), and χ^2^ goodness of fit tests were used as well. The software package R v. 1.8.1 (Venables and Smith 2003) was used for the Poisson Regression analyses.

## Results

A total of 589 Y-tube behavioral assays was conducted. In 25.4% of these, *H. horticola* did not move into either arm of the olfactometer during the 10-minute observation. These inconclusive trials were not included in the results.

### Olfactory response to host eggs and larvae

**Figure 1.**
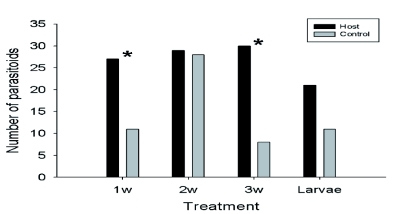
Response of old *Hyposoter horticola* females to one week (1W), two week (2W) and three week (3W) old eggs and newly hatched larvae of their host, *Melitaea cinxia* (Experiment 1). Asterisks denote statistically significant difference in response between the test and control odor, Chi-square, p < 0.05. Note: N° tested *H. horticola*: 60, 83, 50 and 50, respectively; N° non-responding *H. horticola*: 22, 26, 12 and 18, respectively. High quality figures are available online.

Eggs that were 1 and 3 weeks-old were attractive to *H. horticola*^2^_37_ = 6.74, χ^2^_31_ = 12.74, p < 0.05, respectively; Figure 1), but 2 week-old eggs and 1 day-old χ^2^_56_ = 0.02, χ^2^_31_ = 3.13, p > 0.05, respectively; Figure 1). Attraction varied according to *H. horticola* age or experience in the olfactometer. While 1 week-old eggs were most attractive to old *H. horticola* χ^2^_18_ =15.21, p < 0.05), 3 week-old eggs were more attractive to young *H. horticola* χ^2^_20_ = 10.71, p < 0.05).

### Olfactory response to extracts from uninfested plants

**Figure 2.**
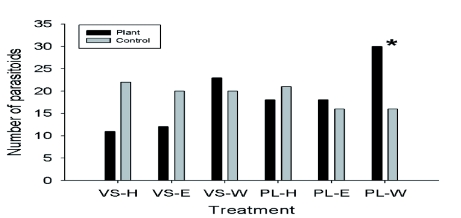
Response of *Hyposoter horticola* to hexane (H), ethanol (E) and water (W) extracts of leaves of *Melitaea cinxia* host plants: *Veronica spicata* (VS) and *Plantago lanceolata* (PL) (Experiment 2). Asterisks denote statistically significant difference in response between the test and control odor, Chi-square, p < 0.05. Note: N° tested *H. horticola* on *V. spicata* in: H = 45, E = 45, and W = 52; and on *P. lanceolata* in: H = 45, E = 45, and W = 52; N° non-responding *H. horticola* on *V. spicata* in: H = 12, E = 13, and W = 9; and on *P. lanceolata* in H = 6, E = 11, and W = 6. High quality figures are available online.

The host plant *P. lanceolata* was attractive χ^2^_45_ = 4.26, p238 = 0.23, p > 0.05 for ethanol, χ^2^_33_ = 0.12, p > 0.05 for hexane; Figure 2). *H. horticola* were not attracted to any of the *V. spicata* χ^2^_42_ = 0.21, p ^2^_32_ = 3.67, p ^2^_32_ = 2.00, p > 0.05; Figure 2).

### Olfactory response to egg-infested plants

**Figure 3.**
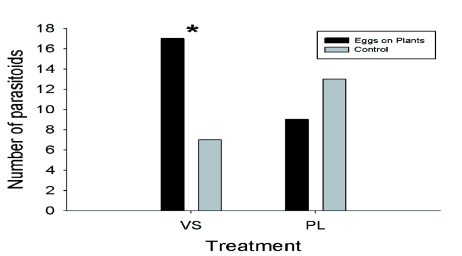
Response of *Hyposoter horticola* to *Veronica spicata* (VS) and *Plantago lanceolata* (PL) harboring eggs of their host, *Melitaea cinxia* (Experiment 3). Asterisks denote statistically significant difference in response between the test and control odor, Chi-square, p < 0.05. Note: N° tested *H. horticola*: 31 and 31, respectively; N° non-responding *H. horticola*: 7 and 9, respectively. High quality figures are available online.

A different pattern emerged when leaves were presented with eggs on them. Overall, *V. spicata* leaves with *M. cinxia* eggs were attractive, but *P. lanceolata* χ^2^_23_ = 4.17, p < 0.05 for *V. spicata*^2^_21_ = 0.73, p > 0.05 for *P. lanceolata*; Figure 3). Further analysis of response to the plants with eggs was done using logistic regression. *H. horticola* age was non-significant. A test of the full model with the three remaining predictors (plant species, egg age, and date) against a constant-only model indicated that the predictors, as a set, reliably distinguished between *H. horticola* χ^2^ (3, *n* = 46) = 221.12, p < 0.0001; Table 1). This analysis showed that the probability of *H. horticola* going to plants with eggs was significantly affected by plant species (z = 4.99; p < 0.0001; Table 1), with *V. spicata* being more attractive, and by egg age (z = 4.91; p < 0.0001; Table 1), with older eggs being more attractive, and by day of test (z = 3.70; p < 0.0001; Table 1).

### Response to host eggs and host plants in a field cage

**Figure 4.**
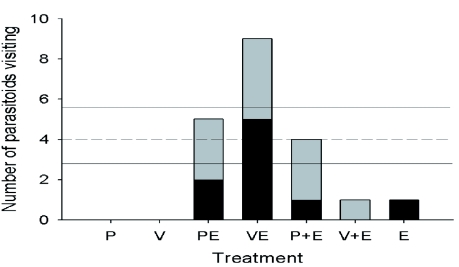
The number of *Hyposoter horticola* finding host egg clusters in each of the treatments in the free-flying parasitoid experiment. The black portion of each bar is the first trial, and the grey portion is the second trial. The dotted line marks the mean number of *H. horticola* individuals visiting, excluding the plants alone (P and V) that were not observed to be visited. The two solid lines indicate the SD of the mean. The visited treatments were *Plantago lanceolata* with eggs on (PE) and next to (P+E) it, *Veronica spicata* with eggs on (VE) and next to (V+E) it, and eggs alone (E). Note that there were twice as many replicates of the eggs alone treatment (E) as each of the other treatments. High quality figures are available online.

Six of the 22 parasitoids released in the first replicate were observed to have found eggs during the two days of observation. In the second replicate, five of the 22 parasitoids were observed to have found eggs during the one day of observation. Figure 4 shows the number of individual parasitoids observed to find eggs in each treatment. Together, there were 31 observations of *H. horticola* at eggs, with some individuals visiting the same or different plants multiple times. Excluding the plants without eggs (that were never observed to be visited by *H. horticola*), each treatment was found by four different parasitoids χ^2^ = 11.28, p = 0.02; Figure 4). Only one *H. horticola* discovered the eggs alone and the eggs next to *V. spicata*, whereas the *V. spicata* with eggs on it was visited by nine of the 11 parasitoids. It is important to note that there were twice as many of the E treatments available to be found. The numbers of *H. horticola* visiting the *P. lanceolata* with eggs on it and eggs next to it were intermediate and not different from the mean. These results indicated that *P. lanceolata* was equally attractive with and without eggs, whereas *V. spicata* was significantly more attractive with eggs on it and unattractive with eggs next to it (Figure 4).

**Table 1.**
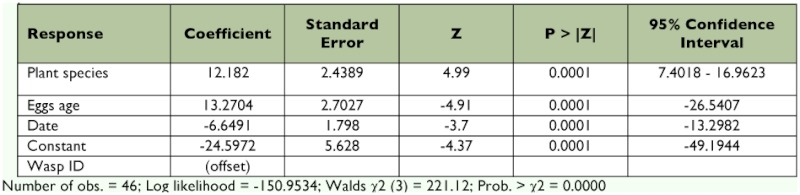
Summary of the logistic regression analysis of the response of *H. horticola* to eggs on plants in the olfactometer experiment.

## Discussion

In this study, a parasitoid's response to components of its foraging environment was observed in two contexts, in an olfactometer and in a large field cage. The larval parasitoid *H. horticola*, which searches for host eggs, was attracted to the odor of eggs in an olfactometer. In the field experiment, however, eggs alone were not sufficiently attractive to be found. In the olfactometer, *H. horticola* responded differently to the two host plant species of *M. cinxia*. *Plantago lanceolata* appeared to be innately attractive, and the presence of host eggs did not increase its attractiveness. In contrast, *V. spicata* became attractive only when host eggs were present. This pattern was reinforced by the results of the field cage experiment, where most *H. horticola* found eggs on *V. spicata*, few found eggs near *V. spicata*, and eggs on and near *P. lanceolata* were found by an intermediate number of *H. horticola*. The results show, for the first time, that a larval parasitoid used egg-induced plant volatiles to find hosts, and that *M. cinxia* eggs or the process of oviposition induced such volatiles.

### Attraction of eggs alone

In the olfactometer, *H. horticola* responded to eggs that were newly laid and eggs that were near hatching, but not to eggs at an intermediate stage. Perhaps initially there is an odor on the eggs that is produced by the adult *M. cinxia*, such as wing scales, sex pheromones, or accessory gland secretion (i.e. Noldus et al. 1991; DeLury et al. 1999; Lian et al. 2007). This odor may subside after several days. Later, a second odor may be perceived by *H. horticola*, perhaps released from the host itself as the embryo develops into a larva. The 2 week-old eggs being apparently undetectable warrants further study. *Hyposoter horticola* should benefit from finding eggs of any age, because it increases the time it has to forage by finding hosts that are not ready for parasitism and monitoring them until they become susceptible (van Nouhuys and Ehrnsten 2004). Therefore, *M. cinxia* that produce non-odorous eggs should have a selective advantage.

Older parasitoids that had been in the olfactometer several times responded to young eggs. Conversely, parasitoids that were young and less experienced were attracted to old eggs. The responses of the parasitoids to foraging cues changes with both wasp age and experience (Vet and Dicke 1992; Papaj and Lewis 1993; Weisser 1994). Unfortunately, the design of the experiment made it unable to distinguish between the two. Regardless of whether *H. horticola* changed behavior due to experience or physiological age, the pattern should be investigated further.

Finally, though *H. horticola* was attracted to the odor of host eggs in the olfactometer, only one wasp found eggs in the field enclosure. The eggs were old, and the parasitoids were young, which meant that the eggs would have been attractive in the olfactometer experiment. This suggests that odor produced by the eggs (or left on the eggs by the mother) did not act as a long-range cue. It may have been too weak or non-volatile to be perceived over distance or in the more complex chemical environment (Hilker and McNiel 2007). The egg odor may instead be useful at a small spatial scale, perhaps arresting *H. horticola* upon alighting on the plant or for locating the egg cluster within the plant.

### Attraction of plants alone

Parasitoids can respond to plant-produced odors even in the absence of an herbivorous host (reviewed by Hilker and McNeil 2007). Chemical components of undamaged *P. lanceolata* and *V. spicata* were extracted in three solvents: water, hexane and ethanol. Strongly polar compounds such as inorganic salts and ionic compounds dissolve only in very polar solvents such as water. Strongly non-polar oils and waxes dissolve only in non-polar organic solvents such as hexane. Ethanol dissolves compounds of intermediate polarity and is a good solvent for most lipids and ionic (inorganic reactives) and non-ionic compounds (organic substrates) (Morrison and Boyd 1998). In the olfactometer, *H. horticola* responded only to the water extract of *P. lanceolata*, which must contain non-volatile or weakly volatile attractants. Somewhat surprisingly, no extract of *V. spicata* was attractive to *H. horticola.*

The low-volatility, high-polarity compounds that can be extracted in water may be short range attractants or contact cues (Jallow et al. 1999; Diongue et al. 2005; Heinz and Feeny 2005) produced by *P. lanceolata* and perceived by *H. horticola*. There is very little information in the literature demonstrating that compounds extracted using water as a solvent are attractive to herbivores or their parasitoids (Tingle and Mitchell 1986; Brown and Anderson 1999; Peterson and Elsey 1995). In contrast, many volatile compounds that are attractive or deterrent to insects have been extracted using low or medium polarity solvents such as hexane and ethanol (i.e., Romeis et al. 1998; Brown and Anderson 1999; Degen et al. 1999; Jallow et al. 1999).

### Eggs and plants together

*H. horticola* forage for eggs that are on plants. They would never experience the odor of eggs alone; the vast majority of host plants do not have eggs on them and presumably are not systematically searched by *H. horticola*. When offered eggs on leaves in the olfactometer, *H. horticola* responded positively only to the *V. spicata*/*egg* combination. The response could have been due to the odor of the eggs, but if that were the case, there should have been some response to the *P. lanceolata*/egg combination. Furthermore, the eggs ranged from 5 to 16 days-ld, putting most of them within the least attractive age. For all of the egg ages, the trend was the same. A more plausible explanation for the difference in response is that the eggs induced *V. spicata* to produce an attractive volatile odor. This has been found in several multitrophic level systems (reviewed by Hilker and Meiners 2006; Fatouros et al. 2008a), but never before for Lepidoptera.

In the field cage experiment, *H. horticola* were not seen on plants lacking eggs on or near them (V and P treatments). This was not surprising because even if they were attracted to plants, they would not be observed because individuals landing on empty plants would have left quickly. Given that the eggs arrest foraging *H. horticola*, the interpretable comparison is among the treatments including eggs. Host eggs next to *P. lanceolata* (P+E) and host eggs on *P. lanceolata* (PE) were found at equal frequency, suggesting that the plant was attractive, but that having eggs on the plant did not make it more attractive. *P. lanceolata* is known to produce volatiles (Fons et al. 1998). Apparently, it produces an airborne odor that is attractive to *H. horticola* and is constitutive rather than induced. This volatile odor is probably not the short range or contact stimulant detected in the olfactometer, which must have had little or no volatility.

*V. spicata* with eggs attached (VE) was frequently found by *H. horticola*, while only one found the eggs next to *V. spicata* (V + E), suggesting, as in the olfactometer experiment, that *V. spicata* changes in response to oviposition. Very little is known about the chemical defense of, and the signaling by, *V. spicata*. However, a second specialized parasitoid of *M. cinxia*, *Cotesia melitaearum* is more attracted to volatiles emitted from herbivore damaged *V. spicata* than from *P. lanceolata* (van Nouhuys and Hanski 2004). Thus for both specialized parasitoids, *V. spicata* is the more attractive host food plant.

### Correspondence of foraging cues with foraging success

In the Åland islands, *P. lanceolata* is present in all suitable habitat patches, as well as in lower densities in unsuitable grassy meadows and roadsides. *Melitaea cinxia* oviposits on only a tiny fraction of plant individuals. In contrast, *V. spicata* is present in a minority of habitat patches and is absent from all non-habitat. Where *V. spicata* is present, *M. cinxia* lays a proportionally higher fraction of eggs clusters on it than on *P. lanceolata* (Kuussaari et al. 2000). Given this unequal distribution of plants, one might expect the opposite pattern of response to host cues than what was found in this study. That is, *H. horticola* would ideally use direct egg-associated cues while searching *P. lanceolata* because there is a high potential for fruitless searching on empty plants, whereas *V. spicata* itself might be a relatively reliable cue.

Based on the results of both the olfactometer and field experiments, the rate of parasitism of hosts on *P. lanceolata* should be lower than on *V. spicata*. However, under natural conditions, *H. horticola* finds virtually all of the egg clusters, and about a third of the larvae in each are parasitized, regardless of which plant species the eggs are laid upon (van Nouhuys and Hanski 2002). In fact, most egg clusters are found by multiple females (van Nouhuys and Ehrensten 2004; van Nouhuys and Kaartinen 2008). This suggests that, though different cues are used for the two host plants, both are sufficient for finding host egg clusters. This contrasts strongly with the other specialist parasitoid, *C. melitaearum*, which experiences metapopulation level effects of differential response to cues associated with *V. spicata* and *P. lanceolata* (van Nouhuys and Hanski 1999).

There are two general conclusions from this study. One is methodological, cautioning the extrapolation of experimental results to explain natural patterns. In particular, interpreting foraging success from observed response to individual foraging cues may be misleading. In this study, *H. horticola* responded to the odor of host eggs in the olfactometer, but in the field cage, that odor alone was insufficient for finding host eggs. Also, *H. horticola* responded quite differently to hosts on one plant species over another in olfactometer experiments and the field cage experiment, but this difference is not reflected in patterns of parasitism that are observed in natural populations.

The more conceptual conclusion is about the expectation of communication between plants and natural enemies of herbivores in multitrophic interactions. Of course, parasitoids of herbivores should use plant-associated cues to find their prey, and it is generally to a plant's advantage for this to occur (Turlings et al. 1995; Kessler and Baldwin 2001; Tscharntke and Hawkins 2002). In this case, speculatively, *V. spicata* may invest more in defense than *P. lanceolata* because *V. spicata* experiences proportionally higher herbivory. Alternatively, if there is competition for resources among plants, and the less abundant *V. spicata* is a poor competitor, it may also invest more in defense. However, even among species that are quite specialized, such as the *P. lanceolata*, *M. cinxia*, *H. horticola* trophic chain, the coupling between a plant and a parasitoid can be weak.

The weak coupling may be expected because the plant would not benefit directly from more reliable host-finding cues. Individual plants do not benefit from parasitism because the parasitized herbivore develops normally until the last instar, and the gregarious larvae consume more than the single plant used for oviposition (Kuussaari et al. 2004). Furthermore, the plant does not need to invest in expensive signals because all egg masses are found (van Nouhuys and Ehrnsten 2004). In this ecological and evolutionary context, and no doubt others, it is perhaps not surprising that parasitoid foraging cues differ among plant species, and that the natural pattern of parasitism is not predicted by *H. horticola* behavior in isolated experiments.
